# Coexisting multi-states in catalytic hydrogen oxidation on rhodium

**DOI:** 10.1038/s41467-021-26855-y

**Published:** 2021-11-11

**Authors:** P. Winkler, J. Zeininger, M. Raab, Y. Suchorski, A. Steiger-Thirsfeld, M. Stöger-Pollach, M. Amati, L. Gregoratti, H. Grönbeck, G. Rupprechter

**Affiliations:** 1 Institute of Materials Chemistry, TU Wien, Getreidemarkt 9, 1060 Vienna, Austria; 2 University Service Center for Transmission Electron Microscopy, TU Wien, Wiedner Hauptstraße 8-10, 1040 Vienna, Austria; 3 Elettra–Sincrotrone Trieste S.C.p.A., SS14 - km 163.5 in Area Science Park, 34149 Trieste, Italy; 4 Department of Physics and Competence Center for Catalysis, Chalmers University of Technology, 412 96 Göteborg, Sweden

**Keywords:** Chemical physics, Surface spectroscopy, Catalytic mechanisms

## Abstract

Catalytic hydrogen oxidation on a polycrystalline rhodium foil used as a surface structure library is studied by scanning photoelectron microscopy (SPEM) in the 10^−6^ mbar pressure range, yielding spatially resolved X-ray photoemission spectroscopy (XPS) measurements. Here we report an observation of a previously unknown coexistence of four different states on adjacent differently oriented domains of the same Rh sample at the exactly same conditions. A catalytically active steady state, a catalytically inactive steady state and multifrequential oscillating states are simultaneously observed. Our results thus demonstrate the general possibility of multi-states in a catalytic reaction. This highly unusual behaviour is explained on the basis of peculiarities of the formation and depletion of subsurface oxygen on differently structured Rh surfaces. The experimental findings are supported by mean-field micro-kinetic modelling. The present observations raise the interdisciplinary question of how self-organising dynamic processes in a heterogeneous system are influenced by the permeability of the borders confining the adjacent regions.

## Abstract (web summary)

Catalytic reactions may exhibit oscillations in the reaction rate even at constant external parameters. Here, the authors observe and explain the coexistence of such oscillations and the steady states of catalytic activity in H_2_ oxidation on differently structured grains of a polycrystalline Rh foil.

## Introduction

Spatiotemporal self-organisation may lead to spontaneous formation of patterns in many biological, chemical and physical systems far from equilibrium^1^. Such patterns have been observed for heart^2^ and brain tissues^3^, liquid crystals^4^, semiconductors^5^ and even for the geographical spread of pandemics^6^ or malware propagation^7^. In chemistry, apart from the well-known Belousov–Zhabotinsky reaction^8^, a wide range of spatiotemporal dynamics has been observed in catalytic surface reactions, such as H_2_ and CO oxidation^9^. Steady-state patterns, travelling waves, moving pulses and oscillating patterns have been detected using surface microscopies, and modelled theoretically^9–12^. Usually, just one type of spatiotemporal behaviour is observed at a time, whereas a coexistence of both steady states and oscillations or travelling waves on the very same sample was not yet observed. However, using a polycrystalline Rh foil consisting of hundreds of µm-sized Rh(hkl) domains, multifrequential oscillations in catalytic H_2_ oxidation were previously detected: the reaction oscillated simultaneously on crystallographically different surface domains exhibiting different frequencies related to the domain structures^13^. On the nanoscale, this phenomenon was also visualised on a Rh curved crystal with the curvature in the range of 10^−3^ nm^−1^, allowing detection of the reconstruction-driven transition from synchronised to multifrequential oscillations along with entrainment, frequency-locking and collapse of spatial coupling^14^.

Using polycrystalline samples and curved crystals allowed establishing the surface structure library concept: surface processes are simultaneously monitored on crystallographically different µm-sized domains or nanofacets of the same sample^15^. This automatically allows identical reaction conditions for all crystallographic orientations, a condition which is hard to fulfil in a conventional “one sample after another” type of measurement^16–18^.

The advantages of surface structure libraries can be exploited only when spatially-resolving methods based on parallel imaging, such as photoemission electron microscopy (PEEM) or field emission/field ion microscopies (FEM/FIM), are applied^19,20^. A limitation with these techniques is that only the local intensity of the recorded image is analysed, whereas chemical sensitivity is lacking. This prevents a straightforward investigation of the atomistic mechanism of the observed effects. In contrast, SPEM, applied in the present work, can—besides imaging the sample surface with sub-micrometre resolution—chemically resolve the surface distribution of individual species via locally resolved XPS (see further details in the Methods section). The use of a technique with chemical information is essential when studying catalytic oxidation reactions on Rh, because the formation of subsurface oxygen and surface oxides may strongly obscure the reaction kinetics^21^.

**Fig. 1:**
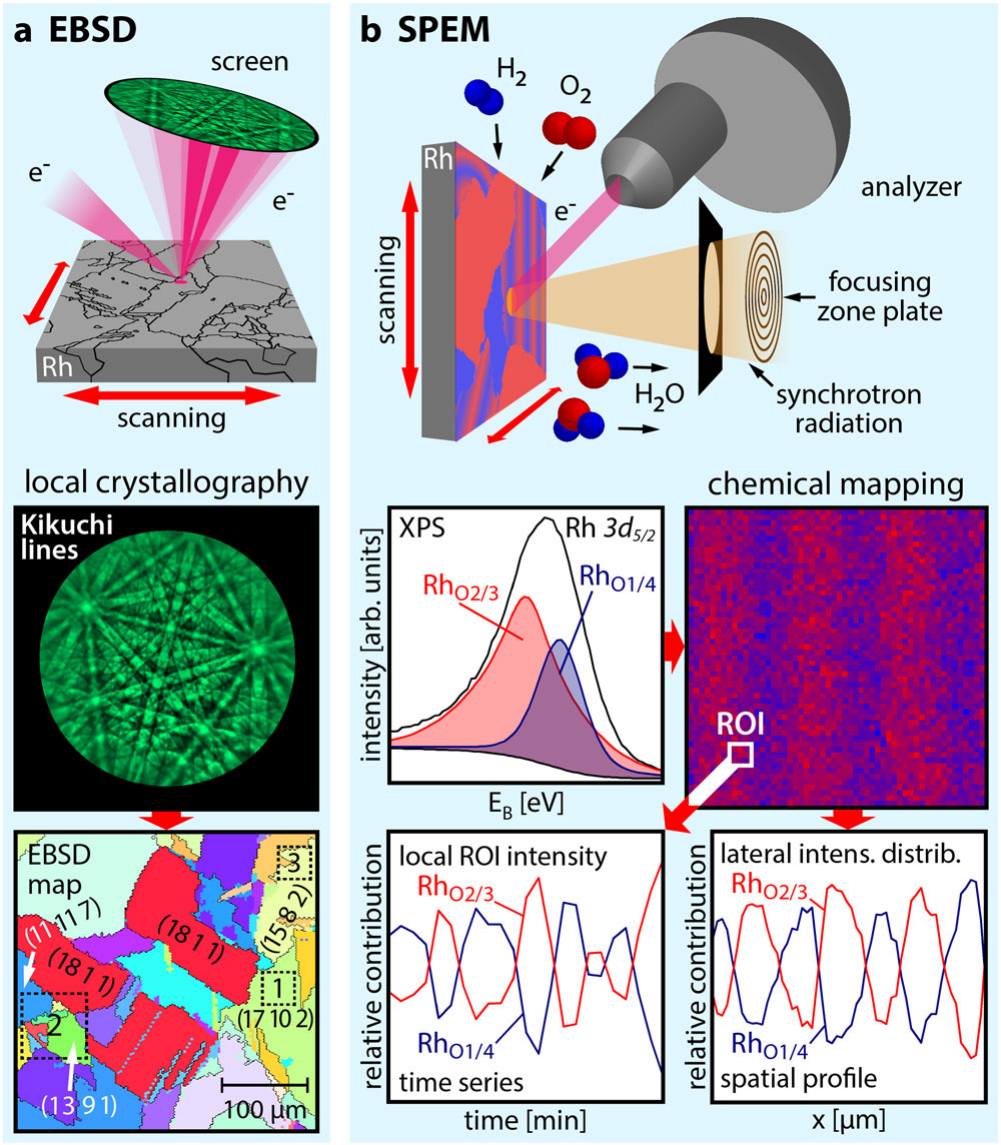
Experimental approach. **a** EBSD: the backscattered electrons of a focused electron beam form Kikuchi lines on a phosphorous screen, enabling determination of the crystallographic orientation of each µm-sized Rh domain; the square regions marked in the EBSD map correspond to those shown in Fig. 2a (region 1) and Fig. 3a (regions 2 and 3); **b** SPEM: the Rh surface is in situ raster-scanned by a sub-µm-sized X-ray spot during H_2_ oxidation, with the emitted photoelectrons providing local XPS spectra. The local adsorbate coverage obtained from the XPS data is displayed as a chemical map. Real-time monitoring reveals both temporal and spatial periodicity of individual spectral components.

The experimental approach is illustrated in Fig. 1: the crystallography of each Rh(hkl) domain of the polycrystalline surface was characterised by electron backscatter diffraction (EBSD; Fig. 1a, see further details in the SI). The ongoing catalytic H_2_ oxidation reaction was visualised in situ by SPEM (Fig. 1b), providing chemical maps, spatial profiles and time series of different spectral components.

In the present work, catalytic hydrogen oxidation is studied by SPEM in the 10^−6^ mbar pressure range on a polycrystalline rhodium foil acting as a surface structure library. Due to the combination of chemical imaging and spatially resolved XPS measurements provided by SPEM, a coexistence of a catalytically active steady state, a catalytically inactive steady state and of multifrequential oscillating states on adjacent domains of the same Rh sample is observed for the first time. This unique behaviour is explained on the basis of the distinguishing behaviour of differently structured Rh surfaces with respect to the mechanism of the kinetic oscillations. The present findings support the formation and depletion of subsurface oxygen as a feedback mechanism for the observed kinetic oscillations. The experimental data are complemented by mean-field micro-kinetic modelling.

## Results and discussion

### Spectromicroscopy of kinetic oscillations

The experiments, in which the SPEM chamber was operated as a flow reactor in the 10^−6^ mbar pressure range, were performed at the “ESCA Microscopy” beamline of the Elettra synchrotron facility^22^.

**Fig. 2:**
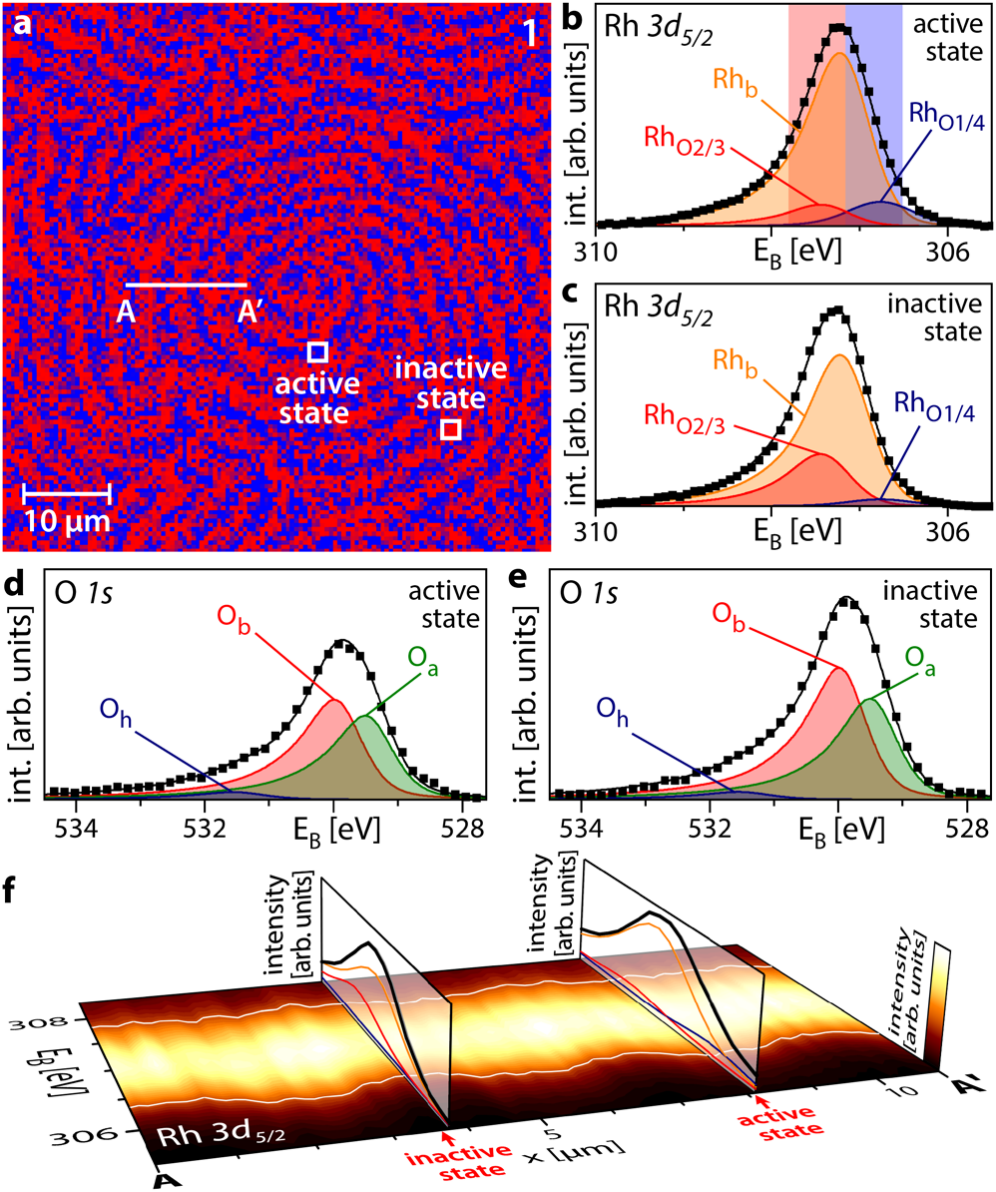
Oscillating pattern in H_2_ oxidation on Rh (T = 453 K, p_O2_ = 1.1 × 10^−6^ mbar, p_H2_ = 1.2 × 10^−^^6^ mbar). **a** Rh *3d*_*5/2*_ SPEM map of region 1 in Fig. 1a showing the distribution of 2/3 (red) and 1/4 (blue) oxygen-bound Rh states on the Rh(17 10 2) domain; **b** Rh *3d*_*5/2*_ XPS spectrum corresponding to the catalytically active state. Rh_b_: bulk Rh; Rh_O2/3_ and Rh_O1/4_ correspond to Rh bound to oxygen; further details are given in the text. Squares: measured values; black solid line: sum of the deconvoluted components. The energy windows for constructing the SPEM map are shaded red and blue; **c** the same as in (**b**) but for the catalytically inactive state; **d** O *1* *s* XPS spectrum corresponding to the catalytically active state. O_a_ and O_b_ are associated with adsorbed atomic oxygen species, while O_h_ corresponds to the OH reaction intermediate. Squares: measured values; black solid line: sum of the deconvoluted components; **e** the same as in (**d**) but for the catalytically inactive state; **f** XPS spectral line profile along the line A-A′ marked in (**a**), with the colour code shown at the right edge. Vertical slices show exemplary spectra for the catalytically inactive and active states. The oscillating white lines serve as a guide for the eye.

Figure 2a shows as an example the Rh *3d*_*5/2*_ SPEM chemical map formed on the Rh(17 10 2) domain (cf. region 1 in the EBSD map in Fig. 1a) during oscillations at T = 453 K, p_O2_ = 1.1 × 10^−6^ mbar and p_H2_ = 1.2 × 10^−6^ mbar. The energy windows (blue and red shaded areas in Fig. 2b) for constructing the map were chosen to reflect different rhodium-oxygen-binding environments. Using reference spectra, these can be related to the state of catalytic activity, as detailed below: the red colour in Fig. 2a corresponds to a catalytically inactive state, while blue corresponds to a catalytically active state. Examples of Rh *3d*_*5/2*_ spectra of both active and inactive surface regions marked in Fig. 2a are given in Fig. 2b, c, respectively.

Deconvolution of the spectra reveals three essential components: Rh_b_, related to bulk rhodium, and Rh_O2/3_ and Rh_O1/4_ which are related to Rh bound to oxygen. In the notation Rh_Oi/j_, i and j refer to the number of O atoms each Rh surface atom is bound to and the number of Rh surface atoms each O atom is bound to, respectively. The deconvolution procedure and notation are based on previous studies, reporting the Rh *3d*_*5/2*_ spectral components characteristic for adsorption of oxygen on different Rh single crystal surfaces^23–26^.

The corresponding O *1* *s* spectra are shown in Fig. 2d, e, where O_a_ and O_b_ correspond to differently adsorbed oxygen species, while O_h_ belongs to the reaction intermediate OH^27,28^. In addition to varying amounts of different oxygen species, the total amount of oxygen (reflected in the total peak area) changes significantly between the two states.

Figure 2f illustrates, using colour-coded photoemission signal intensity, the oscillatory behaviour: the spectral line profile along the line A-A′ marked in Fig. 2a exhibits a sinus-like shape, with the white lines serving as a guide for the eye. Two vertical slices show examples of spectra characterising the catalytically active and inactive states. The switching between catalytically active and inactive states occurs via kinetic transitions, which resemble equilibrium phase transitions, due to the crucial role of cooperative phenomena^29,30^, but take place in a non-equilibrium thermodynamical situation^1,31^. Kinetic transitions in catalytic H_2_ oxidation on Rh were discussed in our previous work^32^.

### Coexisting multistates

**Fig. 3:**
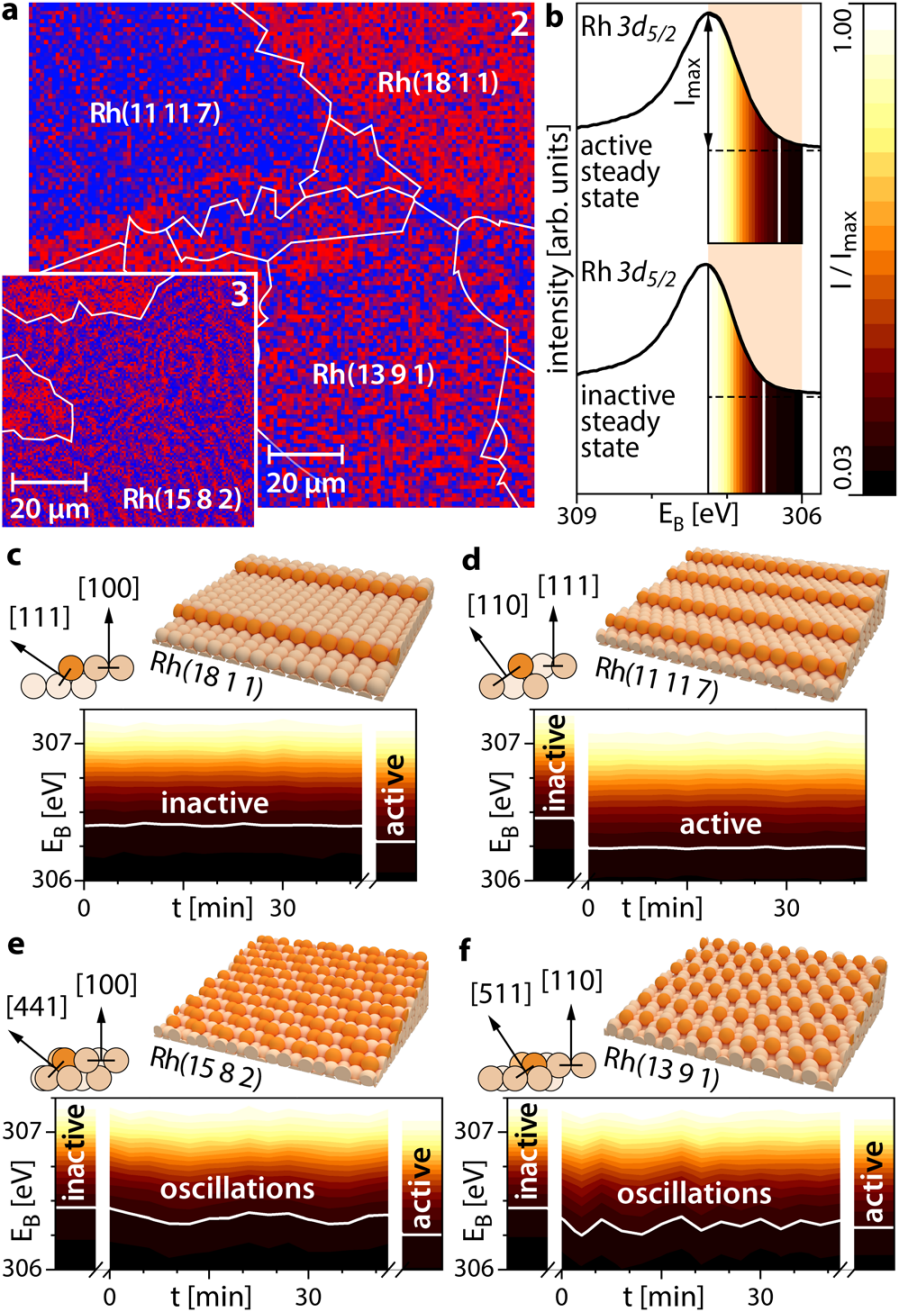
Coexisting multistates in catalytic H_2_ oxidation on Rh. **a** Rh *3d*_*5/2*_ SPEM map of 2/3 (red) and 1/4 (blue) oxygen-bound Rh states of regions 2 and 3 in Fig. 1a during H_2_ oxidation at T = 453 K, p_O2_ = 1.1 × 10^−6^ mbar, p_H2_ = 1.2 × 10^−6^ mbar; **b** reference Rh *3d*_*5/2*_ XPS spectra for the catalytically active and inactive steady states on Rh(13 9 1) at T = 453 K, p_O2_ = 1.1 × 10^−6^ mbar, p_H2_ = 1.6 × 10^−6^ and 0.9 × 10^−6^ mbar, respectively. The XPS signal intensity in the energy window 306–307.25 eV is colour-coded; **c** atomic ball model of the Rh(18 1 1) surface and colour-coded spectral time series in the multi-state regime. The colour-coded active steady state reference spectrum from (**b**) is shown on the right hand side; **d** the same as in (**c**) but for the Rh(11 11 7) surface, with a reference spectrum of the inactive steady state on the left-hand side; e the same as in (**c**) but for the Rh(15 8 2) surface, with two reference spectra on both sides; **f** the same as in (**e**) but for Rh(13 9 1).

The use of a Rh surface structure library allows us to simultaneously visualise the ongoing reaction in situ on Rh(hkl) domains with different atomic surface structures. This is demonstrated in Fig. 3a with Rh *3d*_*5/2*_ chemical maps constructed in the same way as in Fig. 2a, showing regions 2 and 3 from the EBSD map in Fig. 1a. The maps illustrate a unique situation, where different domains of the same sample (and thus at the exactly same p/T conditions) show oscillating patterns on Rh(15 8 2) and Rh(13 9 1), while the Rh(18 1 1) and Rh(11 11 7) domains appear to be entirely in the catalytically inactive and active steady states, respectively. Supplementary Video 1, created from consecutively obtained Rh *3d*_*5/2*_ chemical maps of the Rh(13 9 1) domain, exemplarily shows the oscillating spatiotemporal patterns described above. Further details about the video are given in the SI.

In order to assign the local XPS spectra of a particular domain to a specific state of catalytic activity, reference spectra for the catalytically inactive (at identical T = 453 K, p_O2_ = 1.1 × 10^−6^ mbar and p_H2_ = 0.9 × 10^−6^ mbar) and catalytically active steady states (at the same T and p_O2_ and p_H2_ = 1.6 × 10^−6^ mbar) were acquired for each of the studied domains. Reference Rh *3d*_*5/2*_ spectra for Rh(13 9 1) are exemplarily shown in Fig. 3b with additionally colour-coded XPS signal intensity in the energy window from 306–307.25 eV.

Due to the temporal and spatial periodicity of the oscillation process, time series data can be used in addition to chemically resolved images to probe the oscillating spectral components. Representative spectral time series for the four Rh(hkl) domains mentioned above are shown in Fig. 3c–f using the same colour code for the XPS signal intensity as in Fig. 3b. For reference, spectra of the catalytically inactive and active steady states on the respective Rh(hkl) domain are shown on the sides of the spectral time series. The comparison of Fig. 3c, d again demonstrates that simultaneously some domains remain in the catalytically inactive (e.g. Rh(18 1 1)) or catalytically active (e.g. Rh(11 11 7)) steady states, while others oscillate with differing frequencies (e.g. Rh(15 8 2), f = 0.8 mHz; Rh(13 9 1), f = 2.4 mHz). To our knowledge, this behaviour, i.e. the simultaneous presence of oscillations and both steady states on the same sample at the exactly same external parameters, has not yet been observed for a catalytic surface reaction.

The multiplicity of states observed herein is, however, not a transient state that occurs only temporarily during a kinetic transition, but results from stationary patterns formed under reaction conditions as non-equilibrium (dissipative) structures^33,34^. Since the extension of patterns formed by adsorbates is confined by domain boundaries, such patterns can be treated solely as Turing-like and not as true Turing-structures possessing intrinsic dimensions^35,36^. In addition, the domain boundaries play the role of coherence terminators, disturbing the spatial coupling via hydrogen diffusion which provides the coherence of oscillations within individual domains. This leads to abrupt changes of the oscillation frequencies from one domain to another (frequency transforming) as previously observed^13,37^ or even to a full collapse of the entrainment of the oscillations and their termination as, e.g. in the case of the boundary between Rh(18 1 1) and Rh(13 9 1) visible in Fig. 3a.

This unique behaviour demonstrates the role of the catalyst surface structure in H_2_ oxidation, as illustrated by atomic ball models in Fig. 3c–f: Rh(18 1 1) and Rh(11 11 7), i.e. both domains in the steady state, exhibit step edges of a low-Miller-index type ([111] and [110]), whereas for both oscillating domains, i.e. Rh(15 8 2) and Rh(13 9 1), the step edges include kink sites. Although the presence of certain “atomic roughness” has been previously identified as a prerequisite for the occurrence of oscillations in H_2_ oxidation (oscillations do not occur on a smooth Rh(111) surface^37^), the present study reveals how the atomic structure of the step edges influences the behaviour of particular Rh(hkl) domains.

### Insights into the feedback mechanism

**Fig. 4:**
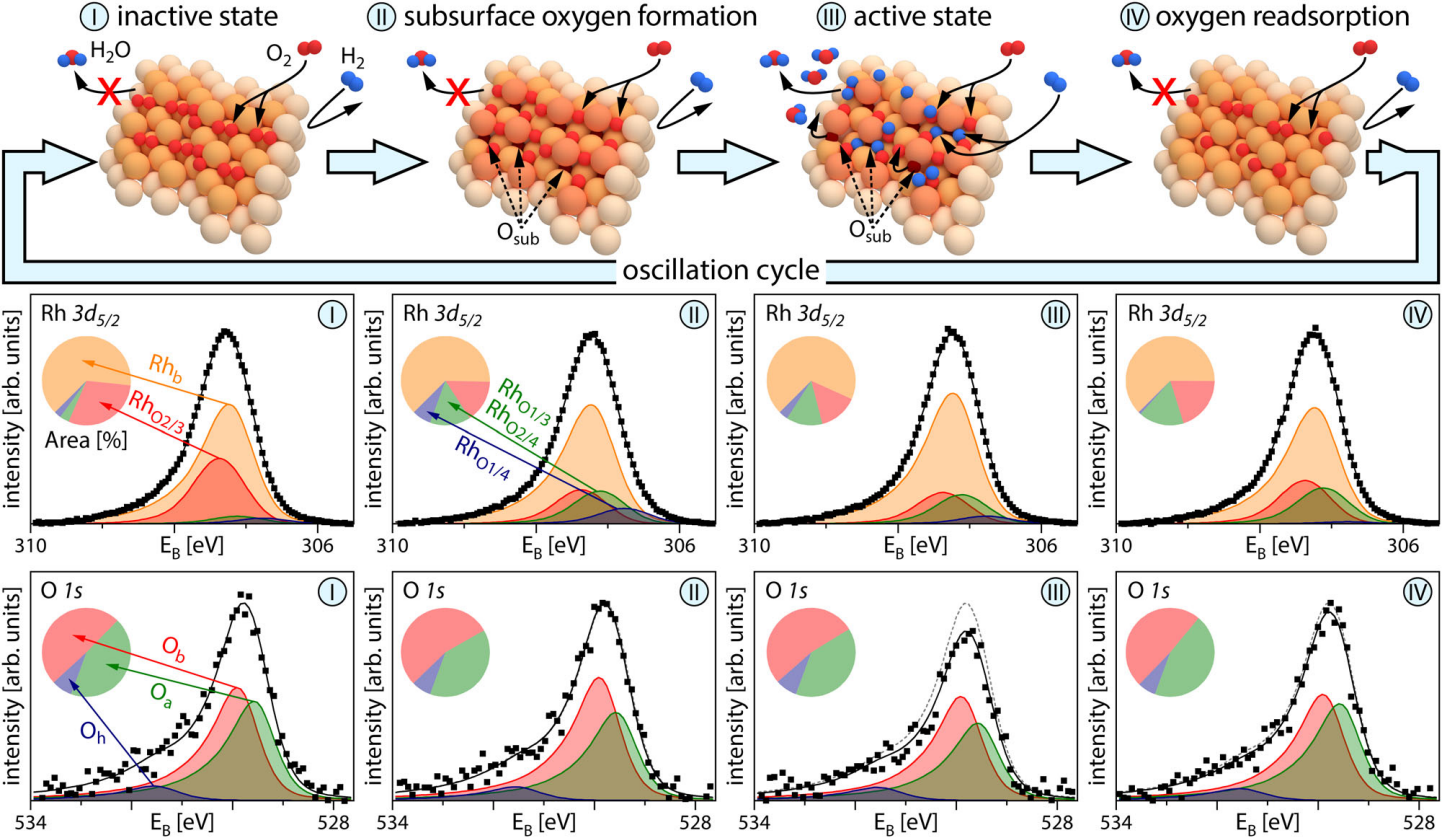
Oscillating components in catalytic H_2_ oxidation on Rh. Upper row: schematic ball models of the four stages of the oscillation cycle, O_sub_ indicates subsurface oxygen species; middle row: deconvoluted Rh *3d*_*5/2*_ XPS spectra obtained during one cycle of oscillations on Rh(15 8 2). The Rh_b_ component relates to bulk Rh, while the Rh_O2/3_, Rh_O1/3_/Rh_O2/4_ and Rh_O1/4_ components correspond to differently oxygen-bound Rh states; further details are given in the text. Squares: measured values; black solid line: sum of the deconvoluted components. The corresponding peak area contributions are given in the pie diagrams; bottom row: deconvoluted O *1s* XPS spectra obtained during one cycle of oscillations. O_a_ and O_b_ components are associated with adsorbed atomic oxygen species, while O_h_ corresponds to the OH reaction intermediate. Squares: measured values; black solid line: sum of the deconvoluted components. The dashed grey lines in steps II–IV indicate the measured data from step I as a reference. The corresponding peak area contributions are given in the pie diagrams.

The presence of coexisting multistates can be rationalised by considering the feedback mechanism governing the oscillations (Fig. 4). The top row in Fig. 4 shows ball models of the four stages of the oscillating cycle, while the middle and bottom rows display corresponding Rh *3d*_*5/2*_ and O *1s* XPS spectra acquired during a single cycle of the oscillations on Rh(15 8 2). As the particular atomic structure of Rh(15 8 2) exhibits occupancies of oxygen binding sites differing from those of the other studied surfaces, a fourth component containing Rh_O1/3_ and Rh_O2/4_, i.e. corresponding to another type of oxygen-bound Rh, was included in the Rh *3d*_*5/2*_ spectra^24^. The pie diagrams show the respective peak area contributions.

The Langmuir–Hinshelwood kinetics of catalytic H_2_ oxidation on Rh, including its oscillating mode, is primarily governed by the adsorption properties of the reactants oxygen and hydrogen^38^. The oscillation cycle starts from the catalytically inactive state (I), characterised by oxygen occupying primarily the energetically favoured threefold-hollow sites^26^. The higher binding energy of oxygen compared to hydrogen^39^ and preferential oxygen adsorption at the step edges^40^ hinder the dissociative adsorption of hydrogen on the step edges^41^, and thus catalytic activity. Due to the resulting dense oxygen coverage, oxygen atoms start to penetrate the Rh surface (stage II of the cycle). The process probably starts at a kink or edge sites which exhibit a larger structural flexibility^42^. While this can be considered the first step towards Rh surface oxidation, the resulting subsurface species should not be considered an ordered surface oxide, which is characterised by a far-ranging O-Rh-O trilayer structure and different XPS spectral signatures^18^. Following this incorporation of oxygen into the surface and the still dense oxygen coverage, some Rh atoms get slightly dislocated, increasing the local surface “roughness”^41^ and changing the binding geometry of adsorbed oxygen^43^. The changes in the binding geometry result in altered Rh *3d*_*5/2*_ spectra, while the total amount of oxygen remains unchanged.

The increased surface “roughness” and the freed threefold-hollow sites create favourable conditions for the dissociative adsorption of hydrogen. Once enough hydrogen is available at the surface, a switch to the catalytically active state (III) takes place, where both hydrogen and oxygen adsorb dissociatively, and hydrogen can diffuse along the step edges and form water via an OH intermediate. This is reflected in a lower amount of oxygen-bound Rh and the corresponding diminished total peak area in the O *1s* spectra. Eventually, also oxygen from subsurface sites will diffuse to surface sites and react. As a result, the rest of the adsorbed oxygen will switch back to the energetically more favourable adsorption sites and oxygen will once again be preferred at the step edges, resulting in a lack of hydrogen supply and the surface switching back to the catalytically inactive state. During the last stage (IV), oxygen at the surface is replenished and the cycle starts again.

Using this description, the peculiarities of the Rh(18 1 1) and Rh(11 11 7) surfaces can also be explained: as already mentioned, the Rh(18 1 1) surface has a relatively low amount of step edges ([111]-type). Due to the kinetic limitations for oxygen penetration into the subsurface^40^, the amount of subsurface oxygen is not sufficient to create surface conditions for dissociative hydrogen adsorption. This domain thus remains in an inactive state. On the contrary, the Rh(11 11 7) surface exhibits plenty of step edges of [110] type, which owing to their structure allow for dissociative hydrogen adsorption at the present conditions. As a result, the Rh(11 11 7) surface maintains its catalytically active state.

While previous studies have provided a few hints about the feedback mechanism based on the formation/depletion of subsurface oxygen^13,37^, the present observations by chemically sensitive SPEM yield the first spectroscopic confirmation of the validity of the subsurface oxygen model.

### Micro-kinetic modelling

To provide a rationale for the observed phenomena, micro-kinetic model simulations based on the Langmuir–Hinshelwood mechanism of H_2_ oxidation on Rh were carried out. The present model version is a field-free-case adaptation of a model originally developed by McEwen et al. to simulate field-induced oscillations^44,45^. The adapted model has already been applied in our previous studies of H_2_ oxidation on Rh^13,37^. In order to account for the role of the step edges in the formation and depletion of subsurface oxygen, the present version was additionally modified to include two different types of sites, namely terrace and step edge sites. The terrace sites are characterised by a higher barrier for the formation and reduction of subsurface oxygen species than the step edge sites. We use the step density resulting from the crystallographic orientation to model the relation of amounts of the two types of sites. Further details on the micro-kinetic model and the used parameters are given in the SI.

**Fig. 5:**
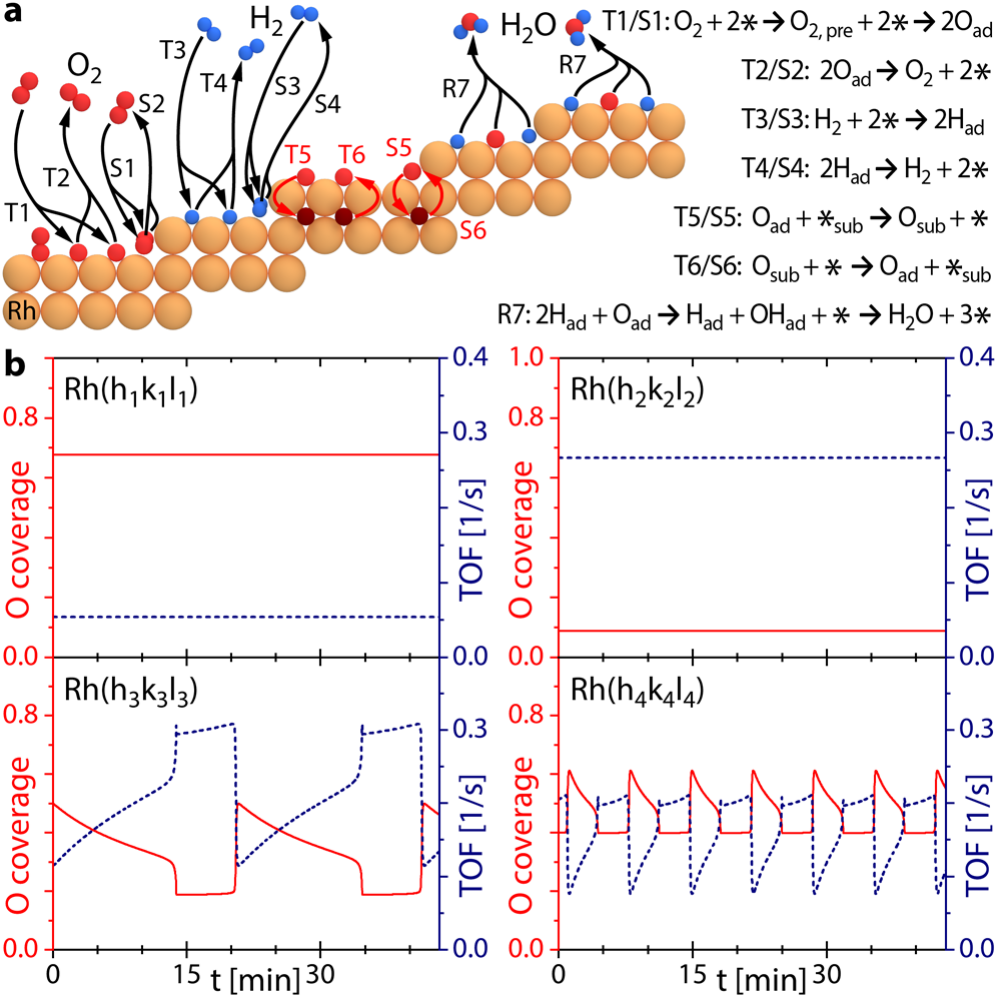
Micro-kinetic simulations of coexisting steady states and oscillations in catalytic H_2_ oxidation on Rh(hkl) domains of different atomic structure for T = 453 K, p_O2_ = 1.1 × 10^−^^6^ mbar, p_H2_ = 1.2 ×10^−6^ mbar. **a** Reaction steps of H_2_ oxidation on a stepped Rh surface included in the micro-kinetic model and corresponding reaction equations, the denominations T and S refer to terrace sites and step edge sites, respectively. The index ad indicates adsorbed species, while the index sub refers to subsurface species and _*_ denotes an empty site; **b** calculated results of the oxygen coverage (red solid line, left ordinate axis) and reaction rate (TOF, blue dotted line, right ordinate axis) for four differently structured Rh surfaces.

Figure 5a schematically depicts the reaction steps considered in the present model, where the letter T refers to terrace sites and the index S to step edge sites: the dissociative adsorption (T1/S1) and associative desorption (T2/S2) of oxygen and hydrogen (T3/S3 and T4/S4), formation (T5/S5) and depletion (T6/S6) of subsurface oxygen species and catalytic water formation (via an OH_ad_ intermediate) and desorption (R7). In contrast to hydrogen, dissociative adsorption of oxygen takes place via a molecular precursor state (T1/S1).

Figure 5b presents the oxygen coverages and reaction rates (turnover frequency; TOF) calculated for the experimental conditions of Fig. 3 for four differently structured Rh(hkl) surfaces, as modelled by terrace and step sites with the corresponding step density. The top left panel shows the results for a surface having [100]-type terraces and 13% non-kinked [111]-type step edges (i.e. similar to the Rh(18 1 1) surface), the top right panel for [111]-type terraces and 39% non-kinked [110]-type step edges (i.e. the Rh(11 11 7) surface), the bottom left panel for [100]-type terraces and 23% highly kinked [110]-type step edges (i.e. the Rh(15 8 2) surface) and the bottom right panel for [110]-type terraces and 50% highly kinked [100]-type step edges (i.e. the Rh(13 9 1) surface). The results of the calculations simulate the experimental behaviour well: the realistic variations of the step density and different basic terrace (i.e. [100]-, [110]- and [111]-structured) and step edge types (i.e. kinked and non-kinked) is sufficient to generate multistates simultaneously present on the differently structured adjacent domains of polycrystalline Rh at identical external parameters.

In summary, the presented study provides an unambiguous demonstration of how the surface structure may influence the local catalytic performance of small surface areas on the very same sample. At certain reaction conditions, adjacent crystallographically different regions of a catalyst are able to coexist in multiple states of catalytic activity. In the present case of catalytic hydrogen oxidation on rhodium, all possible states, i.e. high activity, low activity and multifrequential self-sustained oscillations, were observed simultaneously for µm-sized differently oriented Rh(hkl) domains of a polycrystalline Rh foil. The structure-related peculiarities of the formation and depletion of subsurface oxygen were identified as the key factor for such unusual behaviour. Depending on the specific configuration of terraces and step edges, the rate of formation of subsurface oxygen can either hinder the dissociative adsorption of hydrogen (i.e. the surface is in the catalytically inactive state) on some surfaces or, on other surfaces, promote the hydrogen adsorption (i.e. yielding the catalytically active state). In the range of “intermediate” formation rates of subsurface oxygen, self-sustaining oscillations occur at the same conditions. The present observations support the formation and depletion of subsurface oxygen as a feedback mechanism, which governs the frequency of the oscillations. Mean-field micro-kinetic calculations within a model distinguishing between terrace and step edge surface sites, which differ in their adsorption properties and activation energies for the formation of subsurface oxygen, corroborate the experimental observations.

The observed multistates are made possible because the grain boundaries confining the individual domains are permeable for the propagation of reaction fronts, but still effectively attenuate the spatial coupling. Attenuation of coupling impedes the transfer of a particular state that prevails on one domain to the adjacent regions, i.e. it prevents entrainment effects. Such entrainment was e.g. observed on a curved crystal, where the crystal edges separating different facets are more permeable for reaction fronts than grain boundaries on a polycrystalline sample^14^. Stimulated by the present observations, the interdisciplinary question arises of how the characteristics of borders between adjacent regions, exhibiting different spatiotemporal behaviour, can influence the self-organising dynamic processes in a heterogeneous system.

## Methods

### Preparation and characterisation of the Rh sample

A polished Rh foil (10 × 12 mm², 0.2 mm thickness, 99.99% purity, MaTecK) was used as a polycrystalline Rh sample. The sample was cleaned in UHV by repeated cycles of Ar^+^ ion sputtering at 1 keV at 300 K, annealing to 1073–1173 K and consecutive chemical treatment in oxygen (p_O2_ = 5 × 10^−7^ mbar at 773 K) and hydrogen (p_H2_ = 5 × 10^−6^ mbar at 773 K). The cleanliness of the sample was verified before each experiment and post-experimental analysis routinely performed after SPEM experiments did not indicate any changes in the surface composition.

The foil temperature was measured by a Type K thermocouple spot-welded to its front and regulated by a PID controller within a window of typically 0.25 K. The gas-phase composition was monitored by a mass spectrometer and the reactant partial pressures were constant within measurement accuracy. Previous work has demonstrated the absence of measurable temperature and pressure gradients at the present conditions^46,47^.

Characterisation of the sample crystallography was performed by electron backscatter diffraction (EBSD), providing the crystallographic orientation of each µm-sized domain by scanning the sample surface with a focused electron beam and recording the diffraction patterns generated by the backscattered electrons. EBSD measurements were performed in a field emission scanning electron microscope (FEI Quanta 200 F) using standard EBSD conditions and evaluation procedures^48^, more details are given in the SI.

### Spectromicroscopy of kinetic oscillations

The experiments on the kinetic oscillations of catalytic hydrogen oxidation on Rh were performed with the scanning photoemission microscope (SPEM) hosted at the “ESCA Microscopy” beamline of the Elettra synchrotron facility, which has been described in detail elsewhere^22^. The end station consists of three UHV sub-chambers: the sample is introduced to the system via a fast-entry load lock attached to the first chamber. Using magnetic transfer arms and wobble sticks, the sample can be transferred in UHV to a preparation chamber, which is equipped with facilities for Ar^+^ ion sputtering, annealing, high purity gas supply (H_2_: 99.999%, O_2_: 99.999%) and Auger Electron Spectroscopy (AES) for checking sample composition and cleanliness. Afterwards, the sample is transferred in UHV to the SPEM chamber. A zone plate optical system provides a small focused photon probe (spot diameter 0.13 µm) used to illuminate the sample surface, while the analysed surface region is selected by a piezo specimen positioning and scanning system. The emitted photoelectrons are collected within an 8 eV kinetic energy window by a hemispherical energy analyser equipped with a 48 channel detector.

The SPEM was operated in two modes: in the microspectroscopy mode, an XPS spectrum from a microspot on the sample surface was collected, while in the imaging mode, the sample surface was mapped by synchronised-scanning the sample with respect to the photon probe. In the imaging mode, a 48 points XPS spectrum covering the 8 eV kinetic energy window was recorded for each pixel in the image, allowing determination of the spatial distribution of different chemical species and removal of the topography contribution^49^, and thus creating chemical maps, spatial profiles and time series. A single pixel in a typical SPEM image in the present work takes about 0.05 s to obtain, i.e. the whole image takes below 5 min, while the high-resolution Rh *3d* or O *1s* spectra take less than 1 min. The overall energy resolution is 0.3 eV^50^. Due to the setup geometry, electrons emitted at an angle of 60° to the surface normal were registered. Spectra were taken at a photon energy of 652.75 eV and the energy scales were calibrated against the energy of the Au *4f*_*7/2*_ peak with a binding energy of 84.0 eV. The absence of any drifts in photon energy and photon flux was verified by obtaining Ta *4f* spectra of the chemically inert tantalum clips used for mounting the sample and by obtaining Au *4f* spectra of a separate gold foil sample at regular intervals between the experiments.

All spectra in the present work are representative examples of the described states and were deconvoluted using a pseudo-Voigt line shape^51^ in combination with a Shirley background^52^. The spectral components are based on literature data (see above) and were refined by considering the whole ensemble of spectra, including reference spectra of all clean, catalytically active and catalytically inactive surfaces.

### The micro-kinetic simulations

The micro-kinetic simulations were carried out using a model based on the Langmuir–Hinshelwood mechanism of H_2_ oxidation on Rh. The reaction network included the dissociative adsorption and associative desorption of hydrogen, dissociative adsorption of oxygen via a precursor state and associative desorption of oxygen, formation and depletion of subsurface oxygen and catalytic water formation. In order to account for the role of the step edges in the formation and depletion of subsurface oxygen, in the present model, a distinction was made between two different types of adsorption sites, namely terrace and step edge sites. Details of the model and of the calculations as well as the used calculation parameters are given in the SI.

## Supplementary information

Supplementary Information

Description of Additional Supplementary Files

Supplementary Movie 1

## Data Availability

The data that support the findings of this study are available as zipped folders of annotated HDF4 files and text files in a Zenodo repository under 10.5281/zenodo.5535787^53^.
