# Supplementary material for: Coexisting multi-states in catalytic hydrogen oxidation on rhodium
Source: Nat Commun. 2021 Nov 11;12:6517. doi: 10.1038/s41467-021-26855-y (PMC8586342; doi:10.1038/s41467-021-26855-y)
Supplement: Supplementary file 1 — Supplementary Information [file 41467_2021_26855_MOESM1_ESM.pdf]

Coexisting multi-states in catalytic hydrogen oxidation on rhodium

P. Winkler[1], J. Zeininger[1], M. Raab[1], Y. Suchorski[1], A. Steiger-Thirsfeld[2], M. Stöger-Pollach[2], M. Amati[3], L. Gregoratti[3], H. Grönbeck[4] and G. Rupprechter[1]*

[1]*Institute of Materials Chemistry, TU Wien, Getreidemarkt 9, 1060 Vienna, Austria*
[2]*University Service Center for Transmission Electron Microscopy, TU Wien, Wiedner Hauptstraße 8-10, 1040 Vienna, Austria*
[3]*Elettra–Sincrotrone Trieste S.C.p.A., SS14 - km 163.5 in Area Science Park, 34149 Trieste, Italy*
[4]*Department of Physics and Competence Center for Catalysis, Chalmers University of Technology, 412 96 Göteborg, Sweden*

Supplementary Information

Supplementary Note 1: Determining the crystallographic orientation of Rh(hkl) domains

The determination of the crystallographic orientations of each of the surface domains is crucial when using a polycrystalline sample as a surface structure library [1], as done in the present work. Only then, surface processes such as adsorption, oxidation or ongoing catalytic reactions can be studied with respect to the local surface crystallography and surface structure effects can be uncovered. For samples in the meso-scale range, such as the polycrystalline Rh foil used in this work, electron backscatter diffraction (EBSD) is a suitable technique to determine the crystallographic orientations of each of the studied surface structures.

Based on scanning electron microscopy, EBSD is a well-established crystallographic microstructural characterisation technique, which is commonly used to study crystalline or polycrystalline materials, e.g., in catalysis [2-4], electrochemistry [5-7] or metallurgy [8, 9] and surface oxidation [10]. In the EBSD experiments, a diffraction pattern is formed on a fluorescent screen by the backscattered electrons of an electron beam focused onto a particular sample spot, showing up as so-called Kikuchi lines. As the formed diffraction pattern corresponds to each of the diffracting crystal lattice planes [11, 12], the crystallographic orientation of the particular sample spot can be determined, usually by computer-based image transformation [13, 14]. The concept is routinely applied and has been developed into a standard technique in scanning electron microscopy. In the present study, the EBSD measurements were performed by a FEI Quanta 200F field emission scanning electron microscope using standard EBSD conditions and evaluation procedures [15]. A comparison of pre- and post-reaction EBSD characterisation showed that the surface structures of particular domains remain conserved even after extended periods of ongoing chemical reaction.

The results of the EBSD characterisation are shown as a color-coded map in Supplementary Fig. 1a. The domains are numbered in the order of occurrence in the main text. In Supplementary Fig. 1b, the crystallographic orientations (Miller indices) of the domains are given, and their positions marked on the color-coded inverse pole figure of the cubic lattice (used for colouring the EBSD map in Supplementary Fig. 1a). A Rh *3d*$_{5/2}$ SPEM map of the same region as in Supplementary Fig. 1a is given in Supplementary Fig. 1c, where the contrast results from the sample topography and the differing total photoelectron yield of each of the Rh(hkl) domains. The magnification of all of the used imaging techniques was calibrated by comparing the respective maps with optical micrographs of the same polycrystalline Rh sample.

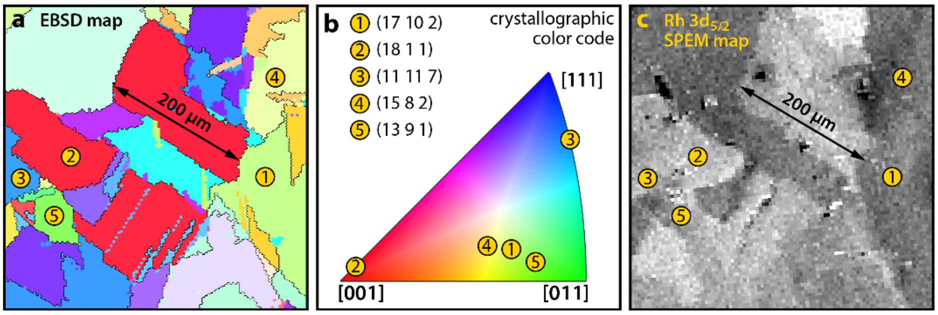

Supplementary Figure 1. Determination of the crystallographic orientations of individual domains of the polycrystalline Rh foil. (a) EBSD color-coded map of the studied region; the individual studied Rh(hkl) domains are numbered; (b) Miller indices of the five Rh(hkl) domains studied in the present work and their location on the inverse pole figure; (c) Rh *3d*$_{5/2}$ SPEM map of the same region as in (a). The image contrast results from the local photoelectron yield.

Supplementary Note 2: Micro-kinetic model simulations

The mean-field microkinetic model applied in our simulations is based on the Langmuir-Hinshelwood mechanism, which is well established for H_2 oxidation on Rh surfaces [16]. The observed kinetic oscillations in this reaction result from the periodic formation/depletion of subsurface oxygen, which influences the adsorption behaviour of both reactants, thus serving as feedback mechanism [4, 17].

The oscillatory water formation is described by seven reactions (Fig. 5: S1/T1 – R7). The formation of water takes place via an OH_{ad} intermediate, where the formation of the intermediate from O_{ad} and H_{ad} is the rate-limiting step in comparison to the subsequent reaction with a second H_{ad} species. Alternative ways of water formation (e.g., by OH disproportionation) can be neglected at the present conditions [18]. We assume that water desorbs immediately after its formation at the considered reaction conditions (453 K), which is justified by the desorption temperature for water being about 300 K on Rh surfaces [19]. The coverages for oxygen (θ_O), subsurface oxygen (θ_S) and hydrogen (θ_H) are in the model described by the following kinetic equations:

$$\frac{\mathrm{d}\theta_\mathrm{O}}{\mathrm{d}t} = \frac{2}{1+K{\theta_*}^2}\left(k_\mathrm{a}^\mathrm{O} K p_{\mathrm{O}_2}{\theta_*}^2 - k_\mathrm{d}^\mathrm{O}{\theta_\mathrm{O}}^2\right) - k_\mathrm{ox}\theta_\mathrm{O}(1-\theta_\mathrm{s}) + k_\mathrm{red}\theta_\mathrm{s}\theta_* - k_\mathrm{r}\theta_\mathrm{H}\theta_\mathrm{O} \quad (1)$$

$$\frac{\mathrm{d}\theta_\mathrm{s}}{\mathrm{d}t} = k_\mathrm{ox}\theta_\mathrm{O}(1-\theta_\mathrm{s}) - k_\mathrm{red}\theta_\mathrm{s}\theta_* \quad (2)$$

$$\frac{\mathrm{d}\theta_\mathrm{H}}{\mathrm{d}t} = 2k_\mathrm{a}^\mathrm{H} p_{\mathrm{H}_2}{\theta_*}^2 - 2k_\mathrm{d}^\mathrm{H}\theta_\mathrm{H}^2 - 2k_\mathrm{r}\theta_\mathrm{H}\theta_\mathrm{O} \quad (3)$$

The empty sites are given by $\theta_* = 1 - \theta_\mathrm{H} - \theta_\mathrm{O}$ and the rate constants by the expressions below, where $\beta = 1/k_B T$. The symbols are explained in Supplementary Tab. 2.

$$k_\mathrm{a}^\mathrm{H} = S_0^\mathrm{H}\, a_\mathrm{s}/\sqrt{2\pi m_{\mathrm{H}_2} k_\mathrm{B} T} \quad (4)$$

$$k_\mathrm{a}^\mathrm{O} = S_0^\mathrm{O}\, a_\mathrm{s}/\sqrt{2\pi m_{\mathrm{O}_2} k_\mathrm{B} T} \quad (5)$$

$$k_\mathrm{d}^\mathrm{H} = k_\mathrm{d0}^\mathrm{O} e^{-\beta E_\mathrm{d}^\mathrm{H}} \quad (6)$$

$$K = K_0 e^{-\beta(E_\mathrm{K} + A_\mathrm{K}^\mathrm{O}\,\theta_\mathrm{O} + A_\mathrm{K}^\mathrm{S}\theta_\mathrm{s})} \quad (7)$$

$$k_\mathrm{d}^\mathrm{O} = k_\mathrm{d0}^\mathrm{O} e^{-\beta(E_\mathrm{d}^\mathrm{O} + A_\mathrm{d}^\mathrm{O}\,\theta_\mathrm{O} + B_\mathrm{d}^\mathrm{O}\,\theta_\mathrm{O}^2)} \quad (8)$$

$$k_\mathrm{ox} = k_\mathrm{ox}^0 e^{-\beta E_\mathrm{ox}} \quad (9)$$

$$k_\mathrm{red} = k_\mathrm{red}^0 e^{-\beta(E_\mathrm{red} + A_\mathrm{red}^\mathrm{S}\theta_\mathrm{s})} \quad (10)$$

$$k_\mathrm{r} = k_\mathrm{r}^0 e^{-\beta(E_\mathrm{r} + A_\mathrm{r}^\mathrm{H}\theta_\mathrm{H} + A_\mathrm{r}^\mathrm{O}\theta_\mathrm{O})} \quad (11)$$

A linear correlation between the activation energies for oxide formation (E_{ox}) and reduction (E_{red}) is assumed [4, 17], based on the results for Rh(100), Rh(110) and Rh(111) reported in Ref. [18], and used to calculate the E_{red} associated with each respective E_{ox} (listed in Supplementary Tab. 2):

$$E_{\mathrm{red}} = 0.293 + 0.776\, E_{\mathrm{ox}} \tag{12}$$

The model applied herein is adapted from a version developed by McEwen et al. to simulate field-induced oscillations in H_2 oxidation on Rh [18, 20, 21]. This model was modified to describe the field-free oscillations on stepped Rh surfaces observed by PEEM [4, 17] and, in the present study, by SPEM. While the original model considers field-induced effects such as a reduction of the activation barrier for oxygen incorporation [22], such effects are not present in our experiments and, therefore, not included in the simulations. The field-free version of the model was already successfully applied to simulate oscillations in the H_2 oxidation on a polycrystalline Rh foil [4, 17] and on a µm-sized curved Rh crystal [23]. Since the present SPEM measurements demonstrated an important role of the step edges in the observed oscillations, we have extended the model in the present work to take effects of steps into account. In particular, the model is modified to include two different site types for each Rh(hkl) domain, namely terrace and step edge sites, as illustrated in Supplementary Fig. 2 for the Rh(18 1 1) and the Rh(11 11 7) surfaces. The two sites are not coupled by diffusion in the modelled reaction kinetics.

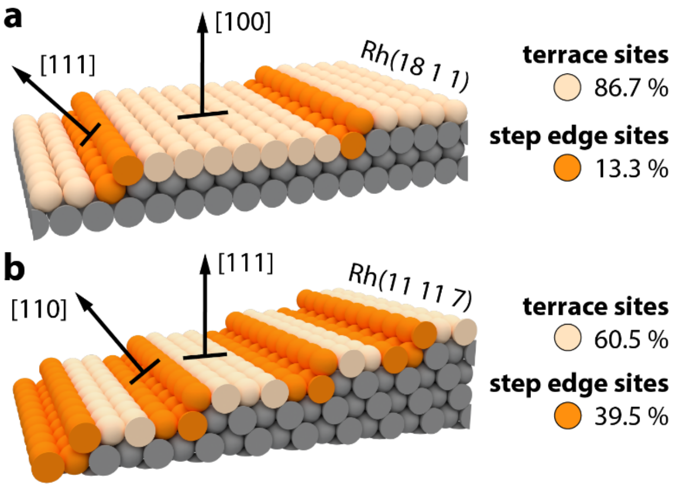

Supplementary Figure 2. (a) Atomic ball model of the stepped Rh(18 1 1) surface with terrace sites and step edge sites coloured in beige and orange, respectively. The bulk atoms are shown in grey; (b) atomic ball model for the Rh(11 11 7) surface. Atomic ball models for the other studied Rh(hkl) surfaces are given in Fig. 3 of the main text.

Each type of sites is assigned a set of kinetic parameters and a set of energy barriers for subsurface oxygen formation E_{ox} and reduction E_{red}. The three used sets of kinetic parameters are based on literature data for Rh(100), Rh(110) and Rh(111) [4, 18] and are listed in Supplementary Tab. 1.

Supplementary Table 1. Parameter sets for Rh(100), Rh(110) und Rh(111) used in the micro-kinetic simulations. Energies are given in eV, the area of a surface site (a_s) in Å^2 and rate constants in 1/s. All simulations have been performed for the same conditions as in the SPEM experiments (T = 453 K, p_{O2} = 1.1 x 10^{-6} mbar, p_{H2} = 1.2 x 10^{-6} mbar). The values are taken from Refs. [4, 18].

symbol	description	(100)	(110)	(111)
a_s	area of a surface site	10	10	10
E_d^H	desorption energy of H	0.75	0.64	0.70
S_0^O	initial sticking coefficient O	0.95	0.95	0.60
A_K^s	coverage dependence of sub-surface oxygen on oxygen dissociation	0.070	0.075	0.082
E_d^O	desorption energy of O	3.5	3.2	2.85
A_d^O	coverage dependence of oxygen desorption energy on adsorbed oxygen	-0.6	-0.5	-0.4
B_d^O	coverage dependence of oxygen desorption energy on molecular oxygen	-0.8	-0.7	-0.5
E_r	activation energy for water formation	0.79	0.79	0.75
S_0^H	initial sticking coefficient H	0.4	0.4	0.4
k_{d0}^h	pre-factor for hydrogen desorption	3.0 x 10^{10}	3.0 x 10^{10}	3.0 x 10^{10}
K_0	pre-factor for oxygen dissociation equilibrium constant	0.2525	0.2525	0.2525
E_k	activation energy for oxygen dissociation equilibrium constant	-0.178	-0.178	-0.178
A_K^O	coverage dependence of adsorbed oxygen on oxygen dissociation	0.158	0.158	0.158
k_{ox}^0	pre-factor for oxygen diffusion from surface to sub-surface sites	5.0 x 10^{11}	5.0 x 10^{11}	5.0 x 10^{11}
k_{red}^0	pre-factor for oxygen diffusion from sub-surface to surface sites	1.85 x 10^{13}	1.85 x 10^{13}	1.85 x 10^{13}
A_{red}^s	coverage dependence of sub-surface oxygen on surface subsurface reduction	0.3	0.3	0.3
k_{d0}^O	pre-factor for oxygen desorption	6.0 x 10^{13}	6.0 x 10^{13}	6.0 x 10^{13}
k_r	pre-factor for water formation	7.0 x 10^{12}	7.0 x 10^{12}	7.0 x 10^{12}
A_r^H	coverage dependence of activation energy of water formation on H	-0.27	-0.27	-0.27
A_r^O	coverage dependence of activation energy of water formation on O	-0.145	-0.145	-0.145

The step edge sites are assigned lower energy barriers $E_{ox,s}$ and $E_{red,s}$ for subsurface oxygen formation and reduction than the corresponding energy barriers for terrace sites $E_{ox,t}$ and $E_{red,t}$. The barriers for the terraces were taken from literature [4, 18]. For kinked step edges (i.e., for the Rh(15 8 2) and Rh(13 9 1) surfaces), $E_{ox,s}$ and $E_{red,s}$ were reduced even further to achieve the experimentally observed oscillations. The energy barriers for oxidation and reduction should be considered as apparent barriers, given that the oxidation and reduction are complex processes at the atomic scale with several elementary steps.
The Miller indices of the stepped domains obtained by EBSD provide crystallographic orientations of the terrace and step edges (Supplementary Tab. 2), which are used for the choice of the kinetic parameter sets and energy barriers. The relative contributions of step edge and terrace sites result from the respective step densities. The chosen energy barriers and relative site type contributions are listed in Supplementary Tab. 2.

Supplementary Table 2. Description of terrace and step edge sites in the micro-kinetic model simulations for individual Rh(hkl) domains. The kinetic parameters associated with Rh(100), Rh(110) and Rh(111) are given in Supplementary Tab. 1; the activation energies for subsurface oxide formation (E_{ox}) are given in eV; the relative contribution of terrace and step edge sites is based on the step density of the domains.

	**terrace sites**			**step edge sites**		
domain orientation	*terrace orientation*	$E_{ox,t}$	*relative contribution*	*main step edge orientation*	$E_{ox,s}$	*relative contribution*
(18 1 1)	(100)	1.520	0.867	(111)	1.300	0.133
(11 11 7)	(111)	1.680	0.605	(110)	1.300	0.395
(15 8 2)	(100)	1.520	0.232	(110)	1.236	0.768
(13 9 1)	(110)	1.630	0.504	(100)	1.174	0.496

Supplementary Note 3: Construction of the Supplementary Video

Supplementary Video 1 shows the spatio-temporal patterns formed during catalytic hydrogen oxidation at T = 453 K, p_{O2} = 1.1 x 10^{-6} mbar and p_{H2} = 1.2 x 10^{-6} mbar on Rh(13 9 1). The video was constructed from chemical maps (41 x 41 μm^2) obtained by SPEM consecutively, which were then used as individual video-frames. For the individual chemical maps, the same energy windows as those in Figs. 2 and 3 in the main text were used, reflecting two different rhodium-oxygen binding environments: $Rh_{O2/3}$ (energy window from 307.16 eV to 307.81 eV) and $Rh_{O1/4}$ (energy window from 306.51 eV to 307.16 eV) [24]. The image contrast in the chemical map results from the relative contribution of the two spectral components, i.e., bright contrast indicates predominance of the $Rh_{O1/4}$ binding geometry, associated with a catalytically active state (cf. Figs. 2 and 3 in the main text), while dark image contrast indicates predominance of the $Rh_{O2/3}$ binding geometry, associated with a catalytically inactive state. Periodic changes between bright and dark stripes in the video illustrate the spatial and temporal periodicity in the adlayer composition and thus in the catalytic activity during the multifrequential self-sustaining oscillations in H_2 oxidation on Rh(hkl) domains.

Supplementary References:

[1] Suchorski, Y. & Rupprechter, G. Heterogeneous Surfaces as Structure and Particle Size Libraries of Model Catalysts. *Catal. Lett.* **148**, 2947–2956 (2018).

[2] Vogel, D. *et al.* The Role of Defects in the Local Reaction Kinetics of CO Oxidation on Low-Index Pd Surfaces. *J. Phys. Chem. C* **117**, 12054–12060 (2013).

[3] Weatherup, R. S. *et al.* In Situ Graphene Growth Dynamics on Polycrystalline Catalyst Foils. *Nano Lett.* **16,** 6196–6206 (2016).

[4] Suchorski, Y. *et al.* Visualizing catalyst heterogeneity by a multifrequential oscillating reaction. *Nat. Commun.* **9**, 600-1–6 (2018).

[5] König, U. & Davepon, B. Microstructure of polycrystalline Ti and its micro-electrochemical properties by means of electron-backscattering diffraction (EBSD). *Electrochim. Acta* **47,** 149–160 (2001).

[6] Yule, L.C. *et al.* Nanoscale electrochemical visualization of grain-dependent anodiciron dissolution from low carbon steel. *Electrochim. Acta* **332**, 135267-1–12 (2020).

[7] Daviddi, E. *et al.* Nanoscale electrochemistry in a copper/aqueous/oil three-phase system: surface structure–activity–corrosion potential relationships. *Chem. Sci.* **12**, 3055-1–15 (2021).

[8] Dingley, D. J. & Randle, V. Microtexture determination by electron back-scatter diffraction. *J. Mater. Sci.* **27,** 4545–4566 (1992).

[9] Schwartz, A. J., Kumar, M., Adams, B. L. & Field, D. P. (eds.) *Electron Backscatter Diffraction in Materials Science.* (Springer US, 2009).

[10] Winkler, P. *et al.* How the anisotropy of surface oxide formation influences the transient activity of a surface reaction. *Nat. Commun.* **12**, 69-1–8 (2021).

[11] Dingley, D. J., Baba-Kishi, K. Z. & Randle V. *Atlas of Backscattering Kikuchi Diffraction Patterns.* (IOP Publishing, 1995).

[12] Baba-Kishi, K. Z. Electron backscatter Kikuchi diffraction in the scanning electron microscope for crystallographic analysis. *J. Mater. Sci.* **37,** 1715–1746 (2002).

[13] Lasse, N.C.K. A New Procedure for Automatic High Precision Measurements of the Position and Width of Bands in Backscatter Kikuchi Patterns. *Mat. Sci. Forum* **273-275**, 201–208 (1998).

[14] Engler, O. & Randle, V. *Introduction to Texture Analysis: Macrotexture, Microtexture and Orientation Mapping*. (CRC Press, Boca Raton, 2009).

[15] Humphreys, F. J. Grain and subgrain characterisation by electron backscatter diffraction. *J. Mater. Sci.* **36,** 3833–3854 (2001).

[16] Zum Mallen, M.P., Williams, W.R. & Schmidt, L.D. Steps in hydrogen oxidation on rhodium: hydroxyl desorption at high temperatures. *J. Phys. Chem.* **97**, 625–632 (1993) and references therein.

[17] Suchorski, Y. *et al.* Surface-Structure Libraries: Multifrequential Oscillations in Catalytic Hydrogen Oxidation on Rhodium. *J. Phys. Chem. C.* **123**, 4217–4227 (2019).

[18] McEwen, J.-S., Gaspard, P., Visart de Bocarmé, T. & Kruse, N. Oscillations and Bistability in the Catalytic Formation of Water on Rhodium in High Electric Fields. *J. Phys. Chem.* **113**, 17045–17058 (2009).

[19] Gregoratti, L. *et el.* Structural effects on water formation from coadsorbed H+O on Rh(100). *Surf. Sci.* **340**, 205–214 (1995).

[20] McEwen, J.-S., Gaspard, P., Visart de Bocarmé, T. & Kruse, N. Nanometric chemical clocks. *Proc. Natl. Acad. Sci.* **106**, 3006–3010 (2009) and SI therein.

[21] McEwen, J.-S., Gaspard, P. & Kruse, N. Electric field induced oscillations in the catalytic water production on rhodium: a theoretical analysis. *Surf. Sci.* **604**, 1353–1368 (2010).

[22] McEwen, J.-S., Gaspard, P., Mittendorfer, F., Visart de Bocarmé, T., & Kruse, N. Field-assisted oxidation of rhodium. *Chem. Phys. Lett.* **452**, 133–138 (2008).

[23] Suchorski, Y. *et al.* Resolving multifrequential oscillations and nanoscale interfacet communication in single-particle catalysis. *Science* **372**, 1314–1318 (2021).

[24] Baraldi, A. *et al.* Spectroscopic Link between Adsorption Site Occupation and Local Surface Chemical Reactivity. *Phys. Rev. Lett.* **93**, 046101-1–4 (2004).
