# Supplementary material for: Coexisting multi-states in catalytic hydrogen oxidation on rhodium
Source: Nat Commun. 2021 Nov 11;12:6517. doi: 10.1038/s41467-021-26855-y (PMC8586342; doi:10.1038/s41467-021-26855-y)
Supplement: Supplementary file 2 — Description of Additional Supplementary Files [file 41467_2021_26855_MOESM2_ESM.pdf]

Description of Additional Supplementary Files

File Name: Supplementary Movie 1

Description: Evolution of a surface chemical pattern on a Rh(13 9 1) domain as obtained by in situ scanning photoelectron microscopy (SPEM) during catalytic hydrogen oxidation at T = 453 K, p_{O2} = 1.1 x 10^{-6} mbar and p_{H2} = 1.2 x 10^{-6} mbar. Field of view of 41 x 41 μm^2.
